# Prevalence of typical circle of Willis and the variation in the anterior communicating artery: A study of a Sri Lankan population

**DOI:** 10.4103/0972-2327.56314

**Published:** 2009

**Authors:** K. Ranil D. De Silva, Rukmal Silva, W. S. L Gunasekera, R. W. Jayesekera

**Affiliations:** 1 Department of Anatomy, Faculty of Medical Sciences, University of Sri Jayewardenepura, Sri Lanka; 2 Department of National Hospital of Sri Lanka; 3 Department of Anatomy, Faculty of Medicine, Colombo

**Keywords:** Anterior communicating artery, anatomical study, circle of Willis, hypoplasia, ischemic stroke

## Abstract

**Objective:**

To determine the extent of hypoplasia of the component vessels of the circle of Willis (CW) and the anatomical variations in the anterior communicating artery (AcomA) in the subjects who have died of causes unrelated to the brain and compare with previous autopsy studies.

**Materials and Methods:**

The external diameter of all the arteries forming the CW in 225 normal Sri Lankan adult cadaver brains was measured using a calibrated grid to determine the occurrence of “typical” CWs, where all of the component vessels had a diameter of more than 1 mm. Variations in the AcomA were classified into 12 types based on Ozaki *et al*., 1977.

**Results:**

193 (86%) showed “hypoplasia”, of which 127 (56.4%) were with multiple anomalies. Posterior communicating artery (PcoA) was hypoplastic bilaterally in 93 (51%) and unilaterally in 49 (13%). Precommunicating segment of the posterior cerebral arteries (P1) was hypoplastic bilaterally in 3 (2%), unilaterally in 14 (4%), and AcomA was hypoplastic in 91 (25%). The precommunicating segment of the anterior cerebral arteries (A1) was hypoplastic unilaterally in 17 (5%). Types of variations in the AcomA were: single 145 (65%), fusion 52 (23%), double 22 (10%) [V shape, Y shape, H shape, N shape], triplication 1 (0.44%), presence of median anterior cerebral artery 5 (2%), and aneurysm 1 (0.44%).

**Conclusion:**

The occurrence of “typical” CW in autopsy brains was rare. Further studies would be necessary to determine if these anatomical variations could predispose to cerebral ischemia and premature stroke in the Sri Lankan population.

## Introduction

Ischemic stroke in young adults (aged 15–45 years) is proportionately more common in India (15–30%)[1] and in Sri Lanka (34%),[2] in contrast to (3–5%)[3] in the West, the etiology of the majority of strokes in young adults in Sri Lanka is unexplained.[4,5]

The circle of Willis (CW) plays an important role in cerebral hemodynamics as a collateral anastomotic channel, and presence of an intact CW should be more effective in facilitating cross flow compared to situations where there are deficiencies in the CW. There is a close correlation between a low capacity CW and an increased risk of stroke,[6–9] collateral ability of the CW be best used when an emergency supervenes, depending on the presence and the size of the luminal caliber of its component vessels.[10–12]

Many studies have repotted a wide range in variation in the anatomy of the CW and the anterior communicating artery (AcomA) among normal individuals,[13–26] hypoplasia of the component arteries of the CW has been studied in India.[17,21,26,27] Hypoplasia of the component arteries of the CW and the anatomical variations of the AcomA has not been previously studied in Sri Lanka and the aim of this cadaveric study was to assess the extent of hypoplasia (diameters <1 mm) of component vessels of the CW; namely the internal carotid arteries (ICAs), precommunicating part of the anterior cerebral arteries (A1), AcomA, precommunicating part of the posterior cerebral arteries (P1), and posterior communicating arteries (PcoA); and the anatomical variations of the AcomA in subjects who have died of causes unrelated to the brain and compare with previous autopsy studies.

## Materials and Methods

225 brains were obtained after ethical approval from medicolegal autopsies on individuals, aged between 18 and 73 years, who had died of causes unrelated to the brain and whose brains demonstrated no gross macroscopic evidence of cerebrovascular disease. The brains were removed from the cranial cavity and fixed in 10% formaldehyde. Blood was carefully washed out from the CW with isotonic saline. The arteries comprising the CW together with the basilar artery with minute branches arising from the main vessels were then carefully removed from the base of the brain. The external diameters of A1, AcomA, PcoA, and P1 were measured using a stereomicroscope equipped with a micrometer-calibrator (Leica). The equipment was standardized according to the manufacturer's specifications. The measurements were performed three times on each segment, by the first author and the calculated average was recorded as the value, line diagrams, and photographic records made, a vessel was recorded as absent only when it was not detected following examination under the dissecting microscope. In the present study, “typical” CW was defined if all of the component vessels of the CW were present, origin of the vessels forming the CW was from its typical source and the size of a component vessel more than 1 mm in diameter.[18] Hypoplasia was defined if a component vessel/s of the CW were less than 1 mm in diameter.[18]

Variations in the AcomA were classified into 12 types: single, one point fusion, long fusion, double, V shape, Y shape, H shape, N shape, triple, plexiform, presence of median anterior cerebral artery, and aneurysms; based on Ozaki *et al*., 1977[23] and compared with studies.[18,22–26] Macroaneurysm of the AcomA were recorded with line diagrams and photographs.

## Results

In the present study, ‘typical circles’ were found only in 32 (14.2%) of the brains. 193 (85.8%) showed hypoplasia, of which 127 (56.4%) were with multiple anomalies. The most frequent site of anomaly was in the posterior half of the circle (70%). There were no instances where any of the component vessels were completely absent.

361 component arteries of the CW were hypoplastic, 255 (70%) posteriorly, and 106 (30%) anteriorly. PcoA was hypoplastic bilaterally in 93 (51.5%), unilaterally in 49 (13.5%), 24 on the left, and 25 on the right side of the CW. P1 was hypoplastic bilaterally in 3 (1.6%), unilaterally in 14 (3.8%), 8 on the left, and 6 on the right side of the CW. AcomA was hypoplastic in 91 (25%). A1 was hypoplastic unilaterally in 15 (4.1%), 5 on the left and 10 on the right side of the CW.

Variations in the AcomA are indicated in Table 1 and Figures 1A–E. Hypoplasia of the AcomA was seen in 91 (25.07%) of the specimens. One (0.44%) macroaneurysm, 25 mm in diameter, was identified arising from the AcomA in a 21-year-old female whose cause of death was homicide [Figure 2].

**Figure 1:**
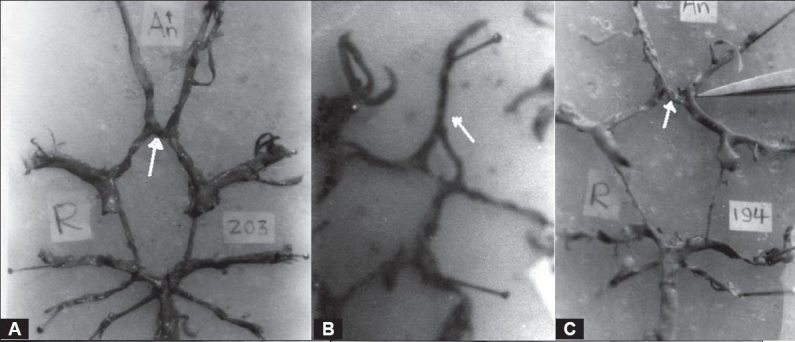
Variations in the AcomA (A) Single, (B) Long fusion, (C) Plexiform

**Figure 1D:**
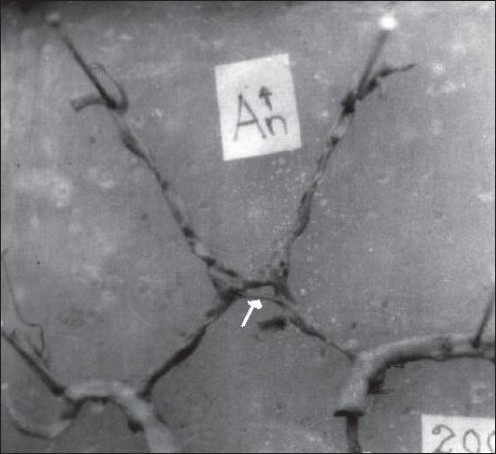
Variations in the AcomA (D) V shape

**Figure 1E:**
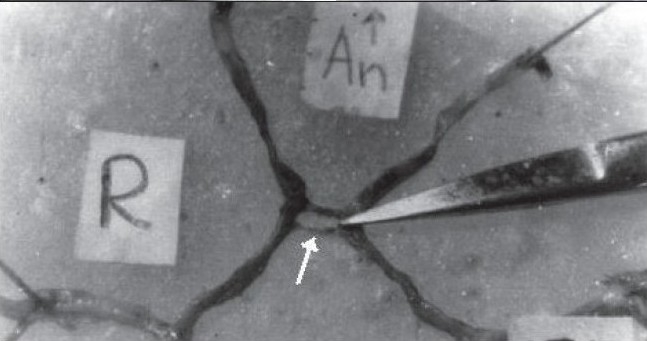
Variations in the AcomA (E) Double

**Figure 2:**
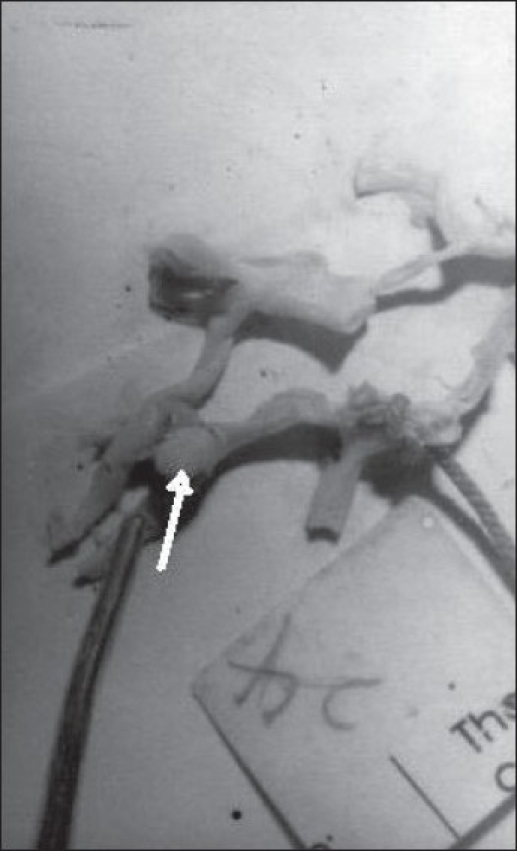
Aneurysm in AcomA

**Table 1:** Comparison of variations in the AcomA as reported in literature

Variation	Fawcett and Blachford 1905[19]	Alpers et al., 1959[18]	Puchades-orts et al., 1976[22]	Ozaki et al., 1977[23]	Fujimoto and Tanaka 1989[24]	Eftekhar et al., 2006[25]	Reddy et al., 1972[21]	Kapoor et al,. 2008[26]	Present Study
Number	700	350	62	148	50			1000	225
Country	UK	USA	Spain	Japan	Japan	Iran	India	India	Sri Lanka
Absence	0.14	2	3.2			1	0.6	1.8	0
Hypoplasia		2.8	6.4			11		2.1	25.07
Single	92.2			39.7	16				65
Fusion				17.6	18				23
Double									
V,Y,H,N shape	7.5,	9	3.2	18.8	28		7	10	10
Triplication	0.14				4			1.2	0.4
Plexiform			4.8					0.4	
Fusion	3.2							
Median ACA		1.7						0.9	2
Aneurysms				2.7				1	0.4

* All variation figures in %

## Discussion

Hypoplasia of the component arteries of the CW has been reported in anatomic studies ranging from 0.7 %[19] to 80.6%,[20] and the proportion of 85.8% observed in the present study, appears to be the highest observed in a population. The prevalence of the ‘typical circle’, the “normal” textbook polygon ranges from 4.6%[28] to 72.2%.[19] A possible reason for the wide range may be the diversity in nomenclature and the criteria used to define hypoplastic vessels. There is little unanimity in nomenclature and quantitative measurement of the diameters of all the component vessels of ‘circle’, which has not been measured in several studies and have relied up on rough estimations of the vessel diameter in determining the anomalies of the CW rather than actual measurements. Vessels have been described as ‘thread-like’, ‘string-like’, ‘minute’, and ‘very small’ without regards to measured diameter.

In the present study, typical configuration was found only in 14.2% of the brains compared to 26.8%,[17] 53.2%,[21] and 45.2%[26] of studies conducted in India and 52.3% in the US.[18] Quantitative measurement of the actual external diameter of all the component vessels of the CW and specimens has been done routinely in the present study. It is believed that Sri Lankans have a common origin from India. The wide range in the prevalence of typical configuration between Indian and Sri Lankan studies, warrants further studies to ascertain influence of genetic, racial, regional, environmental, hemodynamic factors, or a combination of any of them.

The minimum threshold diameter for supplying collateral flow through CW, as assessed by transcranial color-coded duplex ultrasonography (TCCD) and carotid compression tests, was compared with their unfixed postmortem anatomy lies between 0.4 and 0.6 mm. The PcoA threshold diameter for collateral function was slightly higher than the AcomA threshold diameter, possibly due to greater length of PcoA.[29] In the absence of studies showing how far the postmortem arterial diameters of fresh or fixed brains are equal to *in vivo* diameters and the effect of absence of perfusion pressure and possible postmortem shrinking of the arterial wall, in the present study we defined vessels less than 1 mm in diameter as “hypoplastic” or “string-like”.

The most frequent site of abnormal diameters was seen in the posterior half of the circle, and the 70% proportion in the present study is similar to other reported series,[6,18–21,26,30] this may be related to the embryological development of the posterior half of the CW, where the basilar and the ICAs anastomose during development of the cerebral arteries.

Reported incidence of absent arteries in the CW in normal brains leading to an incomplete circle range from 0.6%[18] to 17%.[23] In the present study of 225 autopsies, a vessel was considered absent only when it was not visualized despite careful examination under the dissecting microscope and there were no such instances observed. A meticulous examination is needed to demonstrate small twigs forming the CW. This is dependent upon proper collection of samples, careful removal of the brain and the CW and thorough examination under the dissecting microscope for torn arteries before a vessel is classified as absent. The presence even of small vessels may be important for potential collateral channels.

The state of the circle becomes important in determining the adequacy of the brain circulation. The possibility of by-passing or shunting effects in occlusion of one of the cerebral vessels and the adequacy of recovery or lack of recovery after vascular occlusions may be explained in part by variations in the anatomy of the circle of Willis.[11] A rapid, high reperfusion strongly increases survival in the ischemic penumbra, inhibiting the growth of the core region.

It has been reported that in Asians, the incidence of intracranial atherosclerosis in anterior circulation stroke is much higher than Caucasians.[31] Prevalence of posterior circulation stroke among Asians has been reported much higher compared to the West.[32–34] The average age of patients in the developing countries with stroke is 15 years younger than in developed countries.[35] The reasons for these differences are not well-understood and role of anatomy and the pathology of the cerebral arteries in the pathogenesis of cerebrovascular diseases in different ethnic or racial groups are far from clear. There exist several postulates as to the underlying reasons for the anatomical variation of the CW: amplitude of the neck movements,[36] hemodynamic factors,[15,37] postnatal development,[38] and genetic factors.[39]

The findings of the anomalies of the AcomA in the present study, studies conducted in India[21,26] and from those of more diverse populations,[18,19,21–26] reported in the literature appears to be similar and is possibly due to embryonic development.

### Limitations

In the absence of studies showing relationship between functional *in vivo* diameters and postmortem arterial diameters of fixed brains, we used an arbitrary diameter of 1 mm of component vessels of the CW as hypoplastic. We did not measure the narrowest part of the arteries; these parts probably may determine collateral ability.

### Conclusion

The present study reveals that high incidence of hypoplastic vessels (193 of 225; 86%) and with multiple anomalies (127 of 225; 56.4%), in the CW in the Sri Lankan population, whether it acts in combination with arteriosclerotic changes and/or changes in of food habits and/or genetic difference between normal and anamalous type of cerebral arteries, would contribute to premature strokes in young adults warrants further investigations.
